# Genetic modification of *Anopheles stephensi* for resistance to multiple *Plasmodium falciparum* strains does not influence susceptibility to o’nyong’nyong virus or insecticides, or Wolbachia-mediated resistance to the malaria parasite

**DOI:** 10.1371/journal.pone.0195720

**Published:** 2018-04-10

**Authors:** Andrew Pike, George Dimopoulos

**Affiliations:** W. Harry Feinstone Department of Molecular Microbiology and Immunology, Johns Hopkins Bloomberg School of Public Health, Baltimore, Maryland, United States of America; Instituto Nacional de Salud Pública, MEXICO

## Abstract

Mosquitoes that have been genetically engineered for resistance to human pathogens are a potential new tool for controlling vector-borne disease. However, genetic modification may have unintended off-target effects that could affect the mosquitoes’ utility for disease control. We measured the resistance of five genetically modified *Plasmodium*-suppressing *Anopheles stephensi* lines to o’nyong’nyong virus, four classes of insecticides, and diverse *Plasmodium falciparum* field isolates and characterized the interactions between our genetic modifications and infection with the bacterium *Wolbachia*. The genetic modifications did not alter the mosquitoes’ resistance to either o’nyong’nyong virus or insecticides, and the mosquitoes were equally resistant to all tested *P*. *falciparum* strains, regardless of *Wolbachia* infection status. These results indicate that mosquitoes can be genetically modified for resistance to malaria parasite infection and remain compatible with other vector-control measures without becoming better vectors for other pathogens.

## Introduction

Malaria is a disease of global public health concern that produces millions of cases each year and leads to hundreds of thousands of deaths, largely among children in sub-Saharan Africa [1]. The *Plasmodium* spp. parasites that cause malaria are vectored by various mosquitoes in the genus *Anopheles*, and control of these vector mosquitoes has been employed as part of many malaria control programs. However, traditional vector control methods such as bed nets and insecticides have failed to bring about lasting changes in mosquito populations or reductions in malaria infection levels because of problems such as lack of compliance, difficulties in distribution, and increasing behavioral and physiological resistance in mosquito populations. Therefore, novel vector control methods are constantly being investigated, such the release of transgenic mosquitoes with reduced vector competence or breeding capacity and the use of the endosymbiotic bacterium *Wolbachia* to reduce the ability of the mosquitoes to spread disease [2, 3]. However, despite the creation of numerous transgenic mosquito lines with reduced vector competence in various laboratories, there has been no release of such mosquitoes as part of a coordinated malaria control program, in part because of our lack of knowledge about the interactions of the genetic modifications with other mosquito control technologies.

We have generated several transgenic mosquito lines that suppress malaria parasite infection, based on genetic modification of their innate immune system. Mosquitoes possess an innate immune system capable of responding to various invading pathogens, including bacteria, viruses, and eukaryotic parasites. This immune system comprises multiple pathways, including the Toll and immune deficiency (IMD) pathways, which act to control different types of pathogens. The IMD pathway is responsible for mosquitoes’ resistance to the human malaria parasite *P*. *falciparum* [5], and we have created multiple mosquito lines that transiently over-express the IMD pathway-regulated NF-kB transcription factor Rel2 following a blood meal under the control of the midgut specific carboxypeptidase (CP) promoter or the fat body-specific vitellogenin (VG) promoter [3]. These mosquitoes show greatly increased resistance to *P*. *falciparum* infection in the laboratory, with limited effect on their fitness [3, 4]. We have also previously created mosquito lines transiently over-expressing both a long and a short form of *P*. *falciparum*-specific AgDscam under the control of the CP promoter, and we have shown that they significantly suppress *P*. *falciparum* infection [6].

We have previously tested the global changes in mRNA and protein levels as well as the fitness effects of these modifications and have shown that despite broad changes in gene expression, there are minimal fitness costs attributed to these genetic modifications [7, 4]. However, the strains were initially tested only for resistance against one standard laboratory strain of *P*. *falciparum*.

There are also other considerations in deploying genetically modified mosquitoes. For instance, *Anopheles* mosquitoes also vector o’nyong’nyong virus (ONNV), and other mosquito control methods, including insecticide treatment, are currently or may soon be used, and it is important to understand how these other tools interact with the genetic modification. Knowledge of these interactions will be important for deciding whether or not to utilize genetically modified mosquitoes for malaria control.

Another intervention that has been suggested for the control of vector-borne diseases is the use of the intracellular bacteria *Wolbachia*. This bacteria is a reproductive parasite that uses modification of its host reproductive system to quickly spread to fixation in a population [8]. While not commonly found in many common disease vectors, such as *Aedes aegypti* or the majority of *Anopheles* spp. mosquitoes, it is found in approximately 75% of insect species and numerous other arthropods, showing different reproductive phenotypes depending on its host [9, 10]. Because of its ability to spread in a population, this bacterium was initially suggested as a gene driver to spread a genetically modified mosquito population into a wild-type population [11]. However, later experiments showed that infection with *Wolbachia* can act as an anti-parasite effector by itself, because it reduces infection with various pathogens, including *P*. *falciparum* and multiple viruses in various mosquito species [2, 12, 13]. Therefore, *Wolbachia* is now being considered as a stand-alone disease intervention, and *Wolbachia*-infected *Ae*. *aegypti* are being deployed to combat dengue virus in numerous locations around the world [14]. While *Anopheles* spp. mosquitoes have only recently been infected with *Wolbachia*, and the approach requires further development and improvements before it can be used in malaria control, the mosquitoes do show significantly reduced infection with *P*. *falciparum* [2, 15]. *Wolbachia*-infected *Anopheles* mosquitoes may one day be used to control malaria, as may genetically modified mosquitoes. If this is the case, the two approaches cannot be incompatible, since their distributions may overlap. Likewise, the two interventions could be combined to increase the resistance to *Plasmodium* infection, with *Wolbachia* being used as a gene driver for the genetically modified mosquitoes. However, *Wolbachia* are Gram-negative bacteria, which are generally controlled by the IMD pathway. Therefore, our genetically modified mosquitoes may affect *Wolbachia* infection, which could limit the effectiveness of the intervention [16, 17].

In order to determine the interactions between genetic modifications that render mosquitoes more resistant to the malaria parasite and other mosquito control technologies, we tested the resistance of five genetically modified *An*. *stephensi* lines to multiple *Plasmodium* strains, ONNV, and insecticides, and also assessed the interactions of *Wolbachia* with the genetic modifications. Our studies did not indicate any negative interactions between genetically modified mosquitoes and other mosquito control tools, or an altered susceptibility to ONNV.

## Materials and methods

### Ethics statement

This study was carried out in strict accordance with the recommendations in the Guide for the Care and Use of Laboratory Animals of the National Institutes of Health. The protocol was approved by the Animal Care and Use Committee of the Johns Hopkins University (permit number MO15H144). Commercial anonymous human blood was used for parasite cultures and mosquito feeding, and informed consent was therefore not applicable. The Johns Hopkins School of Public Health Ethics Committee has approved this protocol.

### Mosquito rearing

Wild-type (WT) *An*. *stephensi* (Liston) and transgenic mosquitoes of the CpRel2_15_, VgRel2_1_, CpDsPfs_3_, CpDsPfs_11_, and CpDsPfL_8_ lines (previously known as Cp11, Vg1, Pf-S3, Pf-S11, and Pf-L8, respectively) were reared under standard insectary conditions [3, 6]. Larvae were reared in water-filled trays at low densities and fed ground fish food (Tetra) and cat food pellets (Purina). Adults were maintained on a 12h:12h light:dark cycle at 27°C with 80% humidity and provided constant access to a 10% sucrose solution in water. A review of the environmental monitoring records for the relevant time period shows that temperatures ranged from 26.4–28.2° C and humidity ranged from 79.8–80.5%. Mosquitoes were provided a human blood meal from artificial membrane feeders on warmed water bottles to stimulate egg production. Each generation, GM mosquitoes were screened for the eye fluorescence marker to ensure that all experimental mosquitoes bore the genetic modification. The CpRel2_15_, CpDsPfs_3_, CpDsPfs_11_, and CpDsPfL_8_ lines all bear the EGFP marker under the control of the eye specific 3xP3 promoter, while the VgRel2_1_ line bears the DsRed marker under the same promoter. All five lines contain only a single copy of the transgene [4].

### O’nyong’nyong infections and plaque assays

Adult mosquitoes were infected with ONNV according to standardized procedures [18]. Frozen ONNV stocks were added to baby hamster kidney (BHK) cells and allowed to develop for 48 h; then 83 μl of the supernatant from the cell culture were added to 417 μl of human blood and provided as an infectious meal to 3-day-old adult mosquitoes. The concentration of virus particles included in the blood meals was not measured. However, these methods were previously optimized to infect a majority of WT *An*. *stephensi* mosquitoes. *An*. *stephensi* is not a natural host of ONNV, so a relevant “natural” infection level is not known. After feeding, only fully engorged females were kept for future study. Five days after feeding, blood-fed mosquito midguts were dissected in sterile PBS and ground in 150 μl of DMEM containing 10% FBS and 110 units/mL of penicillin and 110 μg/mL of streptomycin. These samples were used for plaque assays according to standard procedures, and the number of plaque forming units (PFUs) per midgut was recorded 7 days after plating [18]. The median number of PFUs per mosquito midgut was compared using a Kruskal-Wallis test with α = 0.05, while the prevalence of infection, or percent of mosquitoes infected with the virus, was compared with a Fisher’s exact test, also with α = 0.05.

### Insecticide resistance testing

To measure the susceptibility or resistance of the mosquitoes to various insecticides, a standard World Health Organization (WHO) tube assay was performed according to standard procedures [19]. Supplies and insecticide-treated papers were obtained from the WHO. Three- day-old adult mosquitoes were provided a human blood meal from artificial membrane feeders and tested for resistance to insecticides 1 h post-blood meal. Mosquitoes were tested 1 hour after a blood meal to simulate the exposure of a female resting after obtaining a blood meal in nature. Twenty-five blood-fed adult females were exposed to each insecticide for 1 h, and the number of dead mosquitoes was recorded at both 1 and 24 h post-exposure.

### *P*. *falciparum* infections

To determine the mosquitoes’ resistance to various *P*. *falciparum* strains, adult mosquitoes were given infectious blood meals containing gametocytes from the HL1204 or 7g8 strain of *P*. *falciparum* according to standard procedures [3, 6]. Using artificial membrane feeders, 5 day-old adult female mosquitoes were provided a human blood meal containing gametocytes and allowed to feed for 30 min, then maintained under standard insectary conditions until dissection. The HL1204 infectious blood meal contained a gametocytemia of 1.0%, while the 7g8 infectious blood meal contained a gametocytemia of 0.3%. After feeding, fully engorged females were selected and maintained for oocyst enumeration. Seven days after infection, mosquito midguts were dissected into sterile PBS and stained with mercurochrome, and the number of oocysts was counted visually via light microscope. The median number of oocysts per mosquito midgut was compared using a Kruskal-Wallis test, followed by a Dunn’s post-hoc test, using α = 0.05. The prevalence of infection, or percent of mosquitoes infected with the parasite, was compared with a Fisher’s exact test, also with α = 0.05.

### *Wolbachia*-infected mosquito crosses

To determine whether *Wolbachia*-based malaria control strategies are compatible with our genetically modified mosquitoes, we crossed the genetically modified lines with the *Wolbachia*-infected LB1 line from Zhiyong Xi [2]. *Wolbachia* infection was confirmed by polymerase chain reaction to detect the bacterial *wsp* gene [20]. Because the lines are on the same background, we did not perform any backcrosses, but rather tested the *P*. *falciparum* resistance in the offspring of the initial crosses. Control mosquitoes were of two types. *Wolbachia*-uninfected *An*. *stephensi* Liston were used as a *Wolbachia*-negative, non-genetically modified WT control. The first generation offspring of female LB1 mosquitoes mated to WT *An*. *stephensi* Liston males were used as the *Wolbachia*-infected, non-genetically modified LB1 control. Three-day-old adult females were challenged with *P*. *falciparum* as described above. We initially tested infection at a low level by feeding mosquitoes on a diluted gametocyte culture, to mimic the low levels of oocysts found in mosquitoes in nature. However, this low level of infection may mask any additional decrease in infection when the two lines are crossed. Therefore, we repeated the experiment with a higher level of infectious parasites in the blood meal. The low-level infection was carried out with a gametocytemia of 0.05% while the high-level infection was carried out with a gametocytemia of 0.4%. The median number of oocysts per mosquito midgut was compared using a Kruskal-Wallis test, followed by a Dunn’s post-hoc test, using α = 0.05. The prevalence of infection, or percent of mosquitoes infected with the parasite, was compared with a Fisher’s exact test, also with α = 0.05.

## Results and discussion

### Resistance to various *P. falciparum* strains

**Fig 1:**
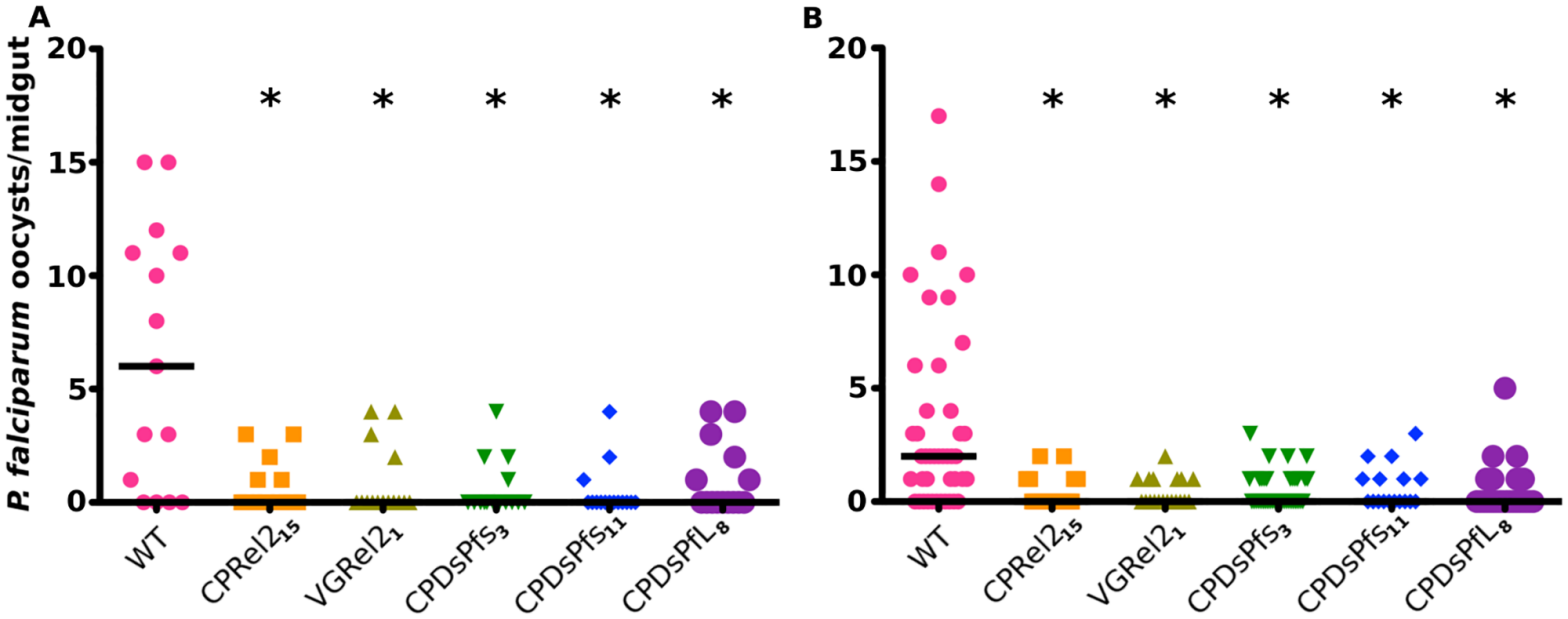
Resistance of genetically modified mosquitoes to multiple *P*. *falciparum* strains. Adult female mosquitoes were fed on infectious blood meals containing gametocytes from the A) Kenyan HL1204 and B) Brazilian 7g8 *P*. *falciparum* strains. All genetically modified mosquito strains tested exhibited increased resistance to both *P*. *falciparum* strains, indicating that these mosquitoes resist *P*. *falciparum* from multiple, geographically divergent, areas. * indicates a significant difference in the number of oocysts per midgut when compared to wild-type mosquitoes using a Kruskal-Wallis test, followed by a Dunn’s post-hoc test and α = 0.05. Additional data are given in S1 Table.

Previous experiments with the genetically modified mosquito strains CpRel2_15_ (expressing the Rel2 transcription factor through the bloodmeal-inducible CP promoter), VgRel2_1_ (expressing the Rel2 transcription factor through the bloodmeal-inducible VG promoter), CpDsPfs_3_ (expressing the AgDscam splice form DsPf-s through the bloodmeal-inducible CP promoter), CpDsPfs_11_ (expressing the AgDscam splice form DsPf-s through the bloodmeal-inducible CP promoter), and CpDsPfL_8_ (expressing the AgDscam splice form DsPf-l through the bloodmeal-inducible CP promoter) only measured resistance to the NF54 line of *P*. *falciparum* [3, 6]. However, there is considerable variation in the ability of diverse *P*. *falciparum* strains to infect specific mosquitoes, so we tested the resistance of these genetically modified mosquitoes against the Kenyan HL1204 and the Brazilian 7g8 strains. All five mosquito lines showed increased resistance to both strains, as measured by parasite oocyst-stage infection of the midgut tissue at 7 days after ingestion of an infectious bloodmeal, indicating that the transgenic mosquitoes can resist diverse strains of *P*. *falciparum* (Fig 1) [21]. It is important to note that, due to low infectivity with these strains, the number of mosquitoes infected and included in this study are limited. Larger studies will more mosquitoes, as well as more additional parasite lines, are important to ensure that these genetic modifications are effective against diverse parasite lineages. However, the results presented here provide a preliminary indication that these mosquitoes are resistant to multiple *P*. *falciparum* lines from various areas.

Studies have shown that the geographic distribution of both mosquito and parasite affect the ability of the parasite to infect the mosquitoes. Here, we have shown that our transgenic mosquitoes are able to resist infection by parasites from different areas [22]. While parasites from a given area might be able to evade the immune response in mosquitoes from other areas, they were not able to do so in our genetically modified mosquitoes. This is likely the result of a broad and strong upregulation of effector genes or their early upregulation after a bloodmeal, which could overwhelm the evasion of the parasites. Our data indicate that these genetically modified mosquitoes represent a viable tool for the development of malaria control strategies, because the same effector mechanism would work in multiple areas against multiple parasite strains.

### Resistance to O’nyong’nyong virus infection

**Fig 2:**
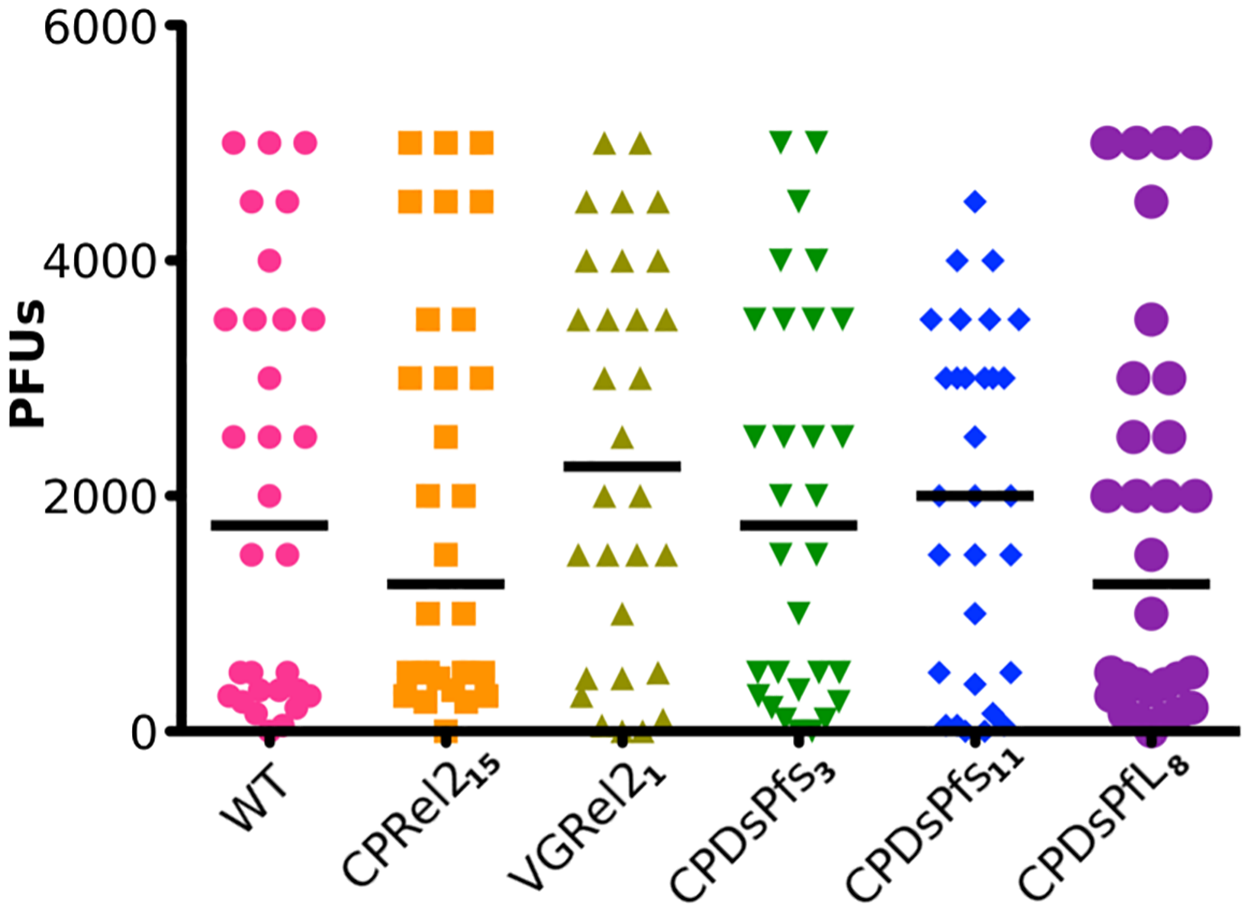
Resistance of genetically modified mosquitoes to ONNV. Adult female mosquitoes were provided an infectious blood meal containing O’nyong’nyong virus (ONNV). Genetically modified mosquitoes showed no difference from wild-type mosquitoes in their ability to be infected with ONNV. This figure represents 10 mosquitoes from each of 3 generations compared by a Kruskal-Wallis test, with α = 0.05. Additional data are presented in S2 Table.

*Anopheles* mosquitoes also act as the vector for the O’nyong’nyong virus, which causes disease symptoms similar to those of dengue fever. This virus has been spreading in recent years [23]. Therefore, in order for genetically modified *Anopheles* mosquitoes to be implemented for malaria control, we needed to confirm that they are unable to spread this virus more efficiently than wild-type mosquitoes. It is also possible that the genetically modified mosquitoes could resist this virus, making them an even more effective vector-borne disease control tool. Therefore, we tested the ability of our transgenic mosquito strains to support ONN infection. Previous studies concerning ONNV infection of mosquitoes have been performed in *An*. *gambiae*, but initial tests in our lab showed that *An*. *stephensi* mosquitoes can also be infected with the virus. Our genetically modified mosquitoes showed no difference in their ability to be infected with ONNV or wild-type mosquitoes, as measured by virus titers in the midgut tissue at 5 days after ingestion of virus (Fig 2). Past studies have not investigated the manner in which *An*. *gambiae* mosquitoes react to or resist ONNV infection, so we do not know the mechanism of resistance. However, other arboviruses, such as dengue virus, are controlled largely by the Toll, JAK/STAT, and RNAi pathways [24–26]. Our mosquitoes show alterations in their IMD pathway activation profile or overexpress *Plasmodium*-specific spliceforms of AgDscam, and therefore it is unlikely that they would be resistant to viruses. However, their lack of increased susceptibility to ONNV reduces the concern that the genetic modification of these mosquitoes could lead to an increase in ONNV transmission.

### Susceptibility to insecticides

**Fig 3:**
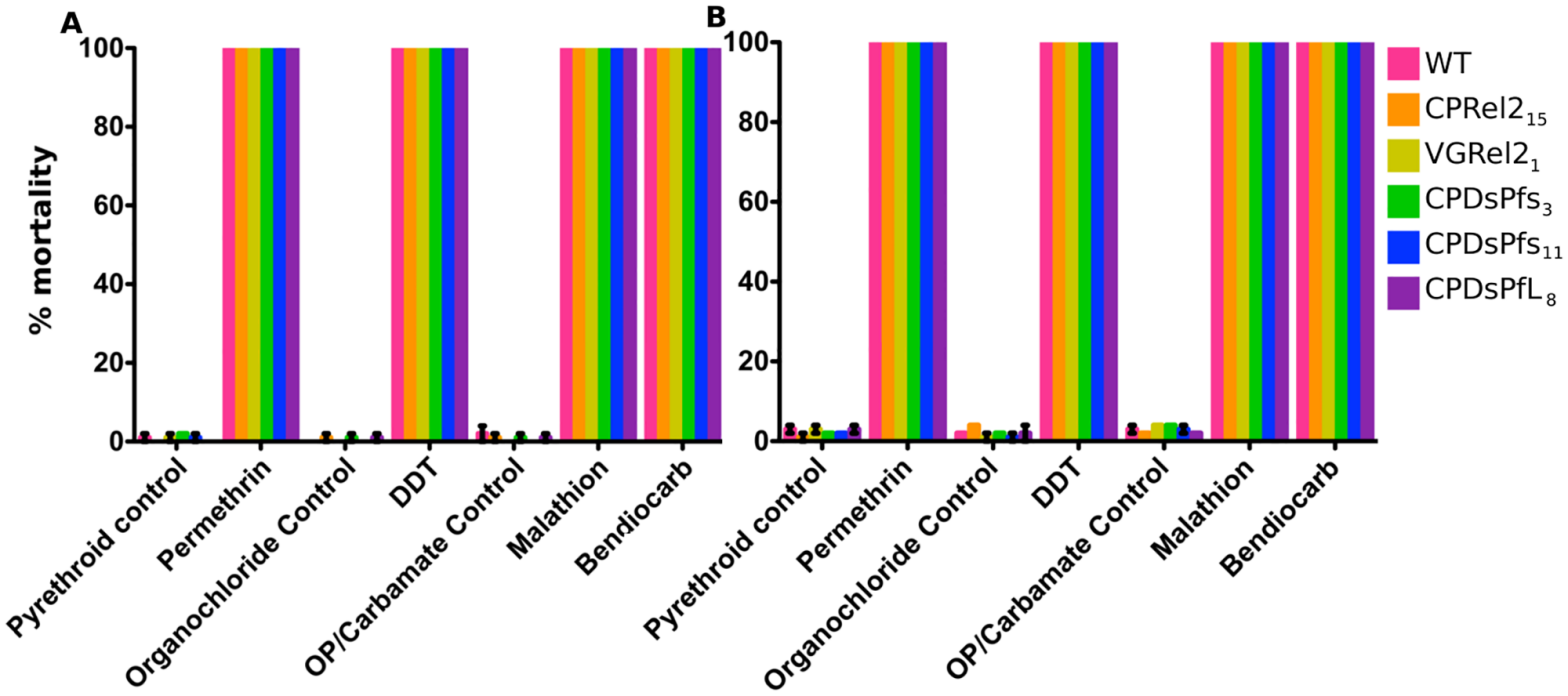
Resistance of genetically modified mosquitoes to insecticides. Bloodfed adult female mosquitoes were exposed to various insecticides for 1 h immediately following a blood meal, and their survival was recorded A) 1 h or B) 24 h post-exposure. Genetically modified mosquitoes showed no difference from wild-type mosquitoes in their resistance to various insecticides. Each figure represents 25 mosquitoes from each of 3 generations.

Insecticides are, and will remain, the primary method of controlling mosquito populations. We tested the susceptibility of our transgenic mosquito strains to insecticides belonging to four commonly used insecticide classes. All five genetically modified lines were exposed to the pyrethroid permethrin, the organophosphate malathion, the carbamate bendiocarb, and the organochloride dichlorodiphenyltrichloroethane (DDT) using a standard WHO assay [27]. Mosquitoes were exposed to the insecticides for 1 hour. At both 1 and 24 h after exposure, all mosquitoes were knocked down, indicating that the genetically modified mosquitoes are as susceptible to insecticides as the wild-type mosquitoes (Fig 3). Control mosquitoes exposed to the carrier oils used to impregnate the test papers with the insecticides showed little or no mortality. The fact that these mosquitoes were highly sensitive to insecticides and that the genetic modifications did not change their resistance to the agents would allow the mosquitoes to be removed from the field if necessary and also to be used in conjunction with long-lasting insecticide-treated bednets or indoor residual spraying. Current vector control methods would not need to be stopped, nor would they lose effectiveness after these genetically modified mosquitoes were released, two results that would likely increase public acceptance of this novel intervention.

### Compatibility with *Wolbachia* infection

**Fig 4:**
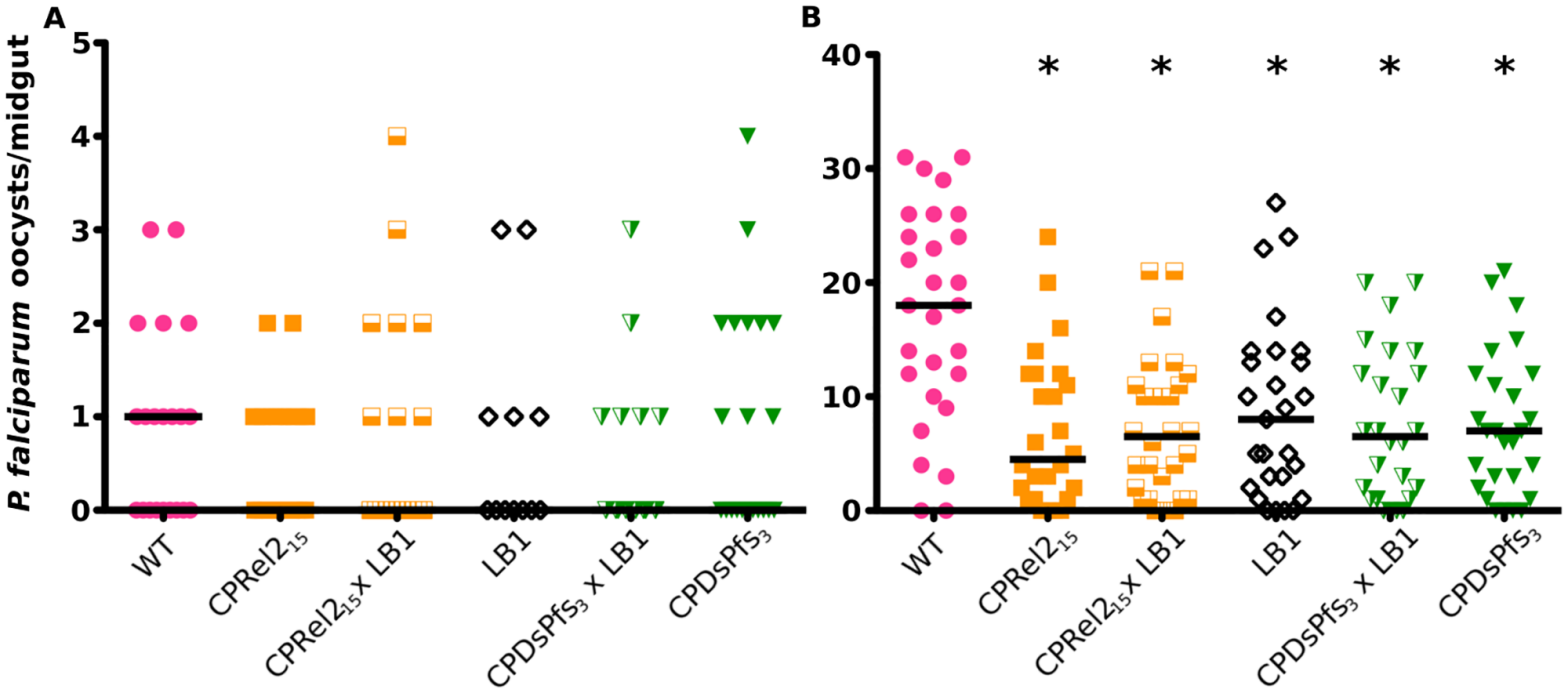
Resistance of genetically modified mosquitoes to *P*. *falciparum* after crossing with *Wolbachia*-infected lines. Genetically modified mosquitoes were crossed with *Wolbachia*-infected mosquitoes to create lines that were both genetically modified and *Wolbachia*-infected. The resulting mosquitoes were provided with a *P*. *falciparum* infectious blood meal at a A) low infection level or B) high infection level. All lines had fewer oocysts on their midguts than did wild-type mosquitoes when infected at a high level, but mosquitoes bearing both the genetic modification and the *Wolbachia* infection did not differ in their *P*. *falciparum* resistance from genetically modified or *Wolbachia*-infected mosquitoes alone, regardless of infection level. Strains were compared using a Kruskal-Wallis test and Dunn’s post-hoc test with α = 0.05. * indicates a significant difference in the number of oocysts per midgut when compared to wild-type mosquitoes. Additional data are given in S3 Table.

We tested the *Plasmodium* resistance of mosquitoes derived from crosses between genetically modified and *Wolbachia*-infected mosquitoes. By crossing virgin *Wolbachia*-infected female mosquitoes with virgin genetically modified male mosquitoes, we created genetically modified mosquitoes that were simultaneously infected with *Wolbachia*. Females of the f1 generation were tested for their resistance to *P*. *falciparum* infection, as measured by oocyst-stage infection of the midgut at 7 days after feeding on malaria-infected blood (Fig 4). We observed no difference in the ability of *Wolbachia*-infected, genetically modified mosquitoes to resist *P*. *falciparum* after single infections at either a low or high infection level. These results indicate that there is no additive effect of combining the two interventions, nor is there a negative effect of combining them. Therefore, the two interventions can be deployed in the same geographic area without hindering resistance to *P*. *falciparum*. As with insecticide susceptibility, the compatibility of genetically modified mosquitoes with *Wolbachia*-infected mosquitoes could be important for the control of *P*. *falciparum*.

## Conclusions

While a variety of genetically modified mosquito strains that show increased resistance to *P*. *falciparum* infection have been created by various research groups, none of these strains has been employed as a part of a widespread malaria control program. All our transgenic mosquito strains suppressed infection with both New and Old World human malaria parasite isolates but showed no increased resistance to ONNV or commonly used insecticides, nor any negative interactions with the bacterium *Wolbachia*. Therefore, these genetic modifications involving the overexpression of the IMD pathway transcription factor Rel2 or *Plasmodium*-specific AgDscam splice-forms can be used in future malaria control programs without negative effects with regard to the tested factors. Although all our experiments were performed in a laboratory setting, none of our evidence suggests that further tests on these lines, including larger cage and field trials, should not be pursued. Prior to wide-scale field releases of the mosquitoes, larger laboratory studies as well as studies under semi-field conditions and small-scale field trials will be important to determine the safety and efficacy of these mosquitoes for vector-borne disease control. The midgut-specific CP promoter-driven Rel2 strains have also been shown to be able to compete with WT mosquitoes and even prevail in a cage population, making this type of genetic modification for *Plasmodium* resistance particularly interesting [4]. Because of our promising data, we conclude that these genetically modified mosquito strains are ready for larger-scale trials.

## Supporting information

S1 Table Supplementary data for Fig 1. The number of mosquitoes assayed, the range, prevalence and median number of oocysts per mosquito midgut. The results of a Kruskal-Wallis Test comparing the median number of oocysts per midgut are presented along with the results of a Dunn’s post-hoc test relative to WT females. The results of the Fisher’s exact test represent the difference in the prevalence of infection, or the number of mosquitoes infected, relative to WT females.
(PDF)

S2 Table Supplementary data for Fig 2. The number of mosquitoes assayed, the range, prevalence and mean in the number of plaque forming units per mosquito midgut. The results of a Kruskal-Wallis Test comparing the median number of PFUs per midgut are provided. The results of the Fisher’s exact test represent the difference in the prevalence of infection, or the number of mosquitoes infected, relative to the WT mosquitoes.
(PDF)

S3 Table Supplementary data for Fig 4. The number of mosquitoes assayed, the range, prevalence, and median number of oocysts per mosquito midgut. The results of a Kruskal-Wallis Test comparing the median number of oocysts per midgut are presented along with the results of a Dunn’s post-hoc test relative to WT or LB1 females, as indicated. The results of the Fisher’s exact test represent the difference in the prevalence of infection, or the number of mosquitoes infected, relative to WT females.
(PDF)
