# Supplementary material for: Genetic modification of Anopheles stephensi for resistance to multiple Plasmodium falciparum strains does not influence susceptibility to o’nyong’nyong virus or insecticides, or Wolbachia-mediated resistance to the malaria parasite
Source: PLoS One. 2018 Apr 10;13(4):e0195720. doi: 10.1371/journal.pone.0195720 (PMC5892925; doi:10.1371/journal.pone.0195720)
Supplement: S1 Table — The number of mosquitoes assayed, the range, prevalence and median number of oocysts per mosquito midgut. The results of a Kruskal-Wallis Test comparing the median number of oocysts per midgut are presented along with the results of a Dunn’s post-hoc test relative to WT females. The results of the Fisher’s exact test represent the difference in the prevalence of infection, or the number of mosquitoes infected, relative to WT females. (PDF) [file pone.0195720.s001.pdf]

S1 Table. Supplementary data for Fig 1.

Figure 1A						
	WT	$CpRel2_{15}$	$VgRel2_{1}$	$CpDsPfs_{3}$	$CpDsPfs_{11}$	$CpDsPfL_{8}$
N	15	15	15	15	15	15
Range	0-15	0-3	0-4	0-4	0-4	0-4
Prevalence	73.33%	33.33%	26.67%	26.67%	20.00%	40.00%
Fisher's test p-value (relative to WT)		0.0656	0.0268	0.0268	0.0092	0.1394
Median	6	0	0	0	0	0
Kruskall-Wallace Test *p*- value	0.0022					
Dunn's Test *p* -value (relative to WT)		≤ 0.01	≤ 0.01	≤ 0.01	≤ 0.001	≤ 0.05
Median no zeroes	10	2	3.5	2	2	2.5
Figure 1B						
	WT	$CpRel2_{15}$	$VgRel2_{1}$	$CpDsPfs_{3}$	$CpDsPfs_{11}$	$CpDsPfL_{8}$
N	43	29	19	38	17	27
Range	0-17	0-2	0-2	0-3	0-3	0-5
Prevalence	66.67%	27.59%	36.84%	39.47%	41.47%	33.33%
Fisher's test *p* -value (relative to WT)		0.0001	0.0092	0.0018	0.0333	0.0011
Median	2	0	0	0	0	0
Kruskall-Wallace Test *p*- value	<0.0001					
Dunn's Test *p* -value (relative to WT)		≤ 0.001	≤ 0.001	≤ 0.001	≤ 0.01	≤ 0.001
Median no zeroes	2.5	1	1	1	1	1
