# Supplementary material for: Genetic modification of Anopheles stephensi for resistance to multiple Plasmodium falciparum strains does not influence susceptibility to o’nyong’nyong virus or insecticides, or Wolbachia-mediated resistance to the malaria parasite
Source: PLoS One. 2018 Apr 10;13(4):e0195720. doi: 10.1371/journal.pone.0195720 (PMC5892925; doi:10.1371/journal.pone.0195720)
Supplement: S2 Table — The number of mosquitoes assayed, the range, prevalence and mean in the number of plaque forming units per mosquito midgut. The results of a Kruskal-Wallis Test comparing the median number of PFUs per midgut are provided. The results of the Fisher’s exact test represent the difference in the prevalence of infection, or the number of mosquitoes infected, relative to the WT mosquitoes. (PDF) [file pone.0195720.s002.pdf]

S2 Table. Supplementary data for Fig 2.

	WT	$CpRel2_{15}$	$VgRel2_{1}$	$CpDsPfs_{3}$	$CpDsPfs_{11}$	$CpDsPfL_{8}$
N	30	30	30	30	30	30
Range	0-5000	0-5000	0-5000	0-5000	0-4500	0-5000
Prevalence	96.67%	96.67%	93.33%	93.33%	93.33%	96.67%
Fisher's test *p* -value		1	0.3311	0.3311	0.3311	1
Median	1500	1250	2000	1750	2250	1250
Kruskall-Wallis Test *p* -value	> 0.05					
