# Supplementary material for: Genetic modification of Anopheles stephensi for resistance to multiple Plasmodium falciparum strains does not influence susceptibility to o’nyong’nyong virus or insecticides, or Wolbachia-mediated resistance to the malaria parasite
Source: PLoS One. 2018 Apr 10;13(4):e0195720. doi: 10.1371/journal.pone.0195720 (PMC5892925; doi:10.1371/journal.pone.0195720)
Supplement: S3 Table — The number of mosquitoes assayed, the range, prevalence, and median number of oocysts per mosquito midgut. The results of a Kruskal-Wallis Test comparing the median number of oocysts per midgut are presented along with the results of a Dunn’s post-hoc test relative to WT or LB1 females, as indicated. The results of the Fisher’s exact test represent the difference in the prevalence of infection, or the number of mosquitoes infected, relative to WT females. (PDF) [file pone.0195720.s003.pdf]

S3 Table. Supplementary data for Fig 4.

Figure 4A						
	WT	$CpRel2_{15}$	$CpRel2_{15}$ x LB1	LB1	$CpDsPfs_3$ x LB1	$CpDsPfs_3$
N	21	22	19	12	14	21
Range	0-3	0-2	0-4	0-3	0-3	0-4
Prevalence	57.14%	40.90%	42.10%	41.67%	42.86%	47.62%
Fisher's test *p* -value (relative to WT)		0.366	0.5273	0.4813	0.4998	0.7579
Median	1	0	0	0	0	0
Kruskall-Wallis Test *p* -value	> 0.05					
Median no zeroes	1	1	2	1	1	2
**Figure 4B**						
	WT	$CpRel2_{15}$	$CpRel2_{15}$ x LB1	LB1	$CpDsPfs_3$ x LB1	$CpDsPfs_3$
N	28	26	30	27	26	27
Range	0-31	0-24	0-21	0-27	0-20	0-21
Prevalence	92.84%	84.62%	86.67%	85.19%	84.62%	85.19%
Fisher's test *p* -value (relative to WT)		0.4126	0.6714	0.4216	0.4126	0.4216
Median	18	4.5	6.5	8	6.5	7
Kruskall-Wallis Test *p* -value	≤ 0.001					
Dunn's Test *p* -value (relative to WT)		≤ 0.001	≤ 0.001	≤ 0.05	≤ 0.01	≤ 0.01
Dunn's Test *p* -value (relative to LB1)	≤ 0.05	> 0.05	> 0.05		> 0.05	> 0.05
Median no zeroes	19	6.5	7	10	7	7
